# The Porcine Odorant-Binding Protein as a Probe for an Impedenziometric-Based Detection of Benzene in the Environment

**DOI:** 10.3390/ijms23074039

**Published:** 2022-04-06

**Authors:** Alessandro Capo, Serena Cozzolino, Adolfo Cavallari, Ugo Bruno, Alessia Calabrese, Angela Pennacchio, Alessandra Camarca, Maria Staiano, Sabato D’Auria, Antonio Varriale

**Affiliations:** 1 Institute of Food Science, CNR Italy, 83100 Avellino, Italy; alessandro.capo@isa.cnr.it (A.C.); se.cozzolino@gmail.com (S.C.); alessia.calabrese@isa.cnr.it (A.C.); angela.pennacchio@isa.cnr.it (A.P.); alessandra.camarca@isa.cnr.it (A.C.); maria.staiano@isa.cnr.it (M.S.); antonio.varriale@isa.cnr.it (A.V.); 2 URT-ISA, CNR at Department of Biology, University of Naples Federico II, 80126 Napoli, Italy; 3 Megaris, Electronics and Electromechanical Systems, 81100 Caserta, Italy; cavallari@megaris.it (A.C.); ugobruno595@gmail.com (U.B.); 4 Department of Biology, Agriculture, and Food Sciences, National Research Council of Italy (CNR-DISBA), Piazzale Aldo Moro 7, 00185 Rome, Italy

**Keywords:** odorant-binding protein (OBP), VOCs, benzene, biosensors

## Abstract

Odorant-binding proteins (OBPs) are a group of small and soluble proteins present in both vertebrates and insects. They have a high level of structural stability and bind to a large spectrum of odorant molecules. In the environmental field, benzene is the most dangerous compound among the class of pollutants named BTEX (benzene, toluene, ethylbenzene, and xylene). It has several effects on human health and, consequently, it appears to be important to monitor its presence in the environment. Commonly, its detection requires the use of very sophisticated and time-consuming analytical techniques (GC-MS, etc.) as well as the presence of specialized personnel. Here, we present the application of an odorant-binding protein (pOBP) isolated from pigs as a molecular recognition element (MRE) for a low-energy impedenziometric biosensor for outdoor and real-time benzene detection. The obtained results show that the biosensor can detect the presence of 64 pM (5 µg/m^3^) benzene, the limit value of exposure for human health set by the European Directive 2008/50/EC.

## 1. Introduction

Odorant-binding proteins (OBPs) are small soluble proteins isolated in the sensory organs of both vertebrates and insects. They are involved in the first steps of olfaction perception [1,2,3]. From a structural point of view, the OBPs of vertebrates belong to the family of lipocalin proteins with a secondary structure organized in eight antiparallel β-sheets and a short α-helical segment close to the C-terminus.

On the contrary, OBPs from insects present a secondary structure with α-helical domains.

The common characteristics of these two classes of OBP are their extreme stability to the exposure to high temperatures and organic solvents. These features make OBPs suitable to be used in different biotechnological applications, such as molecular recognition elements (MREs) in biosensor development or as scavengers for pollutants and other harmful compounds [4].

Recently, we explored the use of the porcine odorant-binding protein (pOBP) to develop a competitive fluorescent assay for benzene detection [5].

In this work, we explored the application of the pOBP for the development of an impedenziometric biosensor for the outdoor and real-time detection of benzene in the environment. The advantages of this kind of biosensor are low energy consumption, small dimensions compared to the classical methods for benzene detection, and the simplicity of performing the measurements [6,7]. In particular, we have derivatized the surface of a gold interdigitated electrode (IDEs) via the thiol chemistry approach [8,9,10,11,12], and then functionalized it with the pOBP. The sensor was tested for its capability to detect benzene in solution and directly in air. The measured impedance is the sum of all the individual contributions, and it is usually described by ohmic resistance, capacitance, and phase [13]. The pOBP is immobilized on the working electrode, and when it interacts with the benzene, it is possible to monitor a change in the capacitance value. For the benzene recognition in air, we developed a specific gas-generated chamber. The obtained results showed that our sensor is able to detect the presence of benzene in the air at a concentration of 64 pM (5 µg/m^3^).

## 2. Results

**Figure 1:**
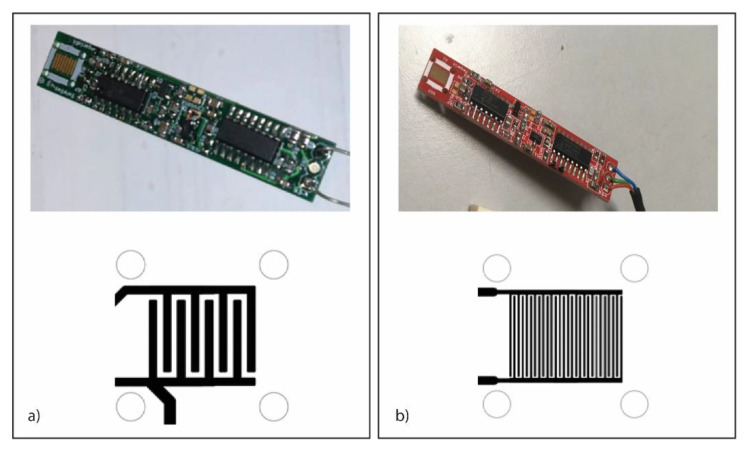
Gas sensor. Photo (on **top**) and scheme of sensing area (on **bottom**) of the gas sensor prototypes with a gold-plated IDE at 200 µm (**a**) and 75 µm (**b**).

Figure 1 shows the structures of interdigitate impedenziometric electrodes. The two PCB surfaces show the interdigitated electrodes in different finger spacing, such as at 200 µm (Figure 1a) and 75 µm (Figure 1b).

**Figure 2:**
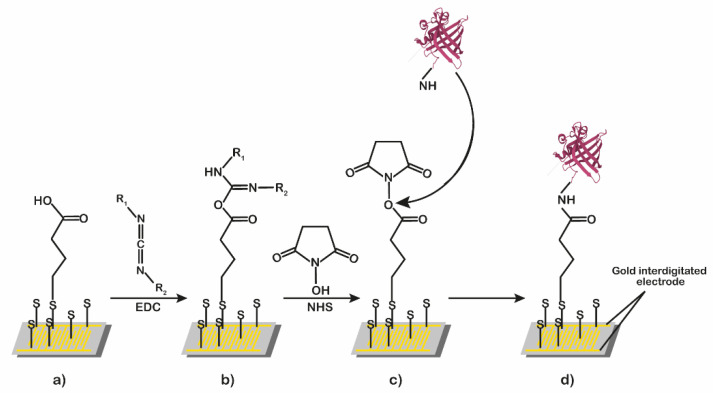
Schematic representation of surface derivatization and functionalization process. The gold surface derivatized with α-lipoic acid (**a**) was treated sequentially with a mixture of EDC/NHS (**b**), and then with a solution of pOBP (**c**) or GlnBP (**d**).

Figure 2 shows the processes of surface derivatization and functionalization. The gold surface was derivatized with α-lipoic acid (Figure 2a), and then it was treated sequentially with a mixture of EDC/NHS (Figure 2b,c), with a solution of the pOBP or glutamine-binding protein (GlnBP) used as the blank reference (Figure 2d) [14].

### 2.1. Benzene Measurement on Interdigitated 200 µm Thin Functionalized Chip

**Figure 3:**
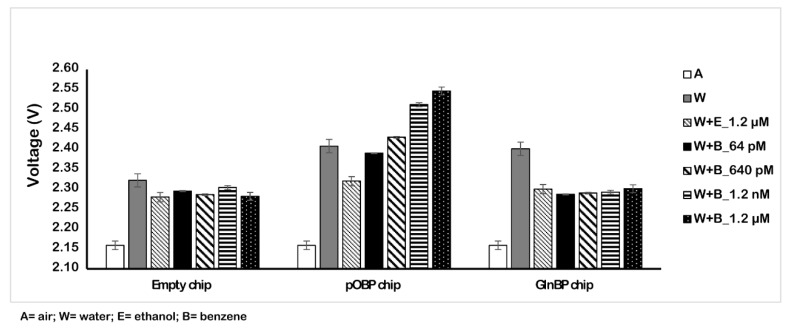
Sensor measurements in solution. Variation of the voltage in the absence (dry, water, and ethanol) and in the presence of different concentrations of benzene (64 pM and 1.2 µM) by using the 200 µm sensor chip.

Firstly, we tested the binding capability of the functionalized chip (200 µm) to detect benzene in solution. Figure 3 displays the obtained results. In particular, we registered the variation of the voltage value in the absence (dry, water, and ethanol) and in the presence of different concentrations of benzene (ranging from 64 pM to 1.2 µM). The results demonstrate the binding of benzene on the chip surface that is registered as an increase of the voltage value. Table 1 reports the voltage values in three different conditions (water, ethanol, and benzene) and, from the dry conditions, we observed an increase of 387 mV at the maximum concentrations of benzene (1.2 µM). The variation of the voltage value (calculated as the difference between the two surfaces (pOBP-GlnBP)), together with the combined standard deviation error analysis, allow us to distinguish the tested benzene concentration in solution.

**Figure 4:**
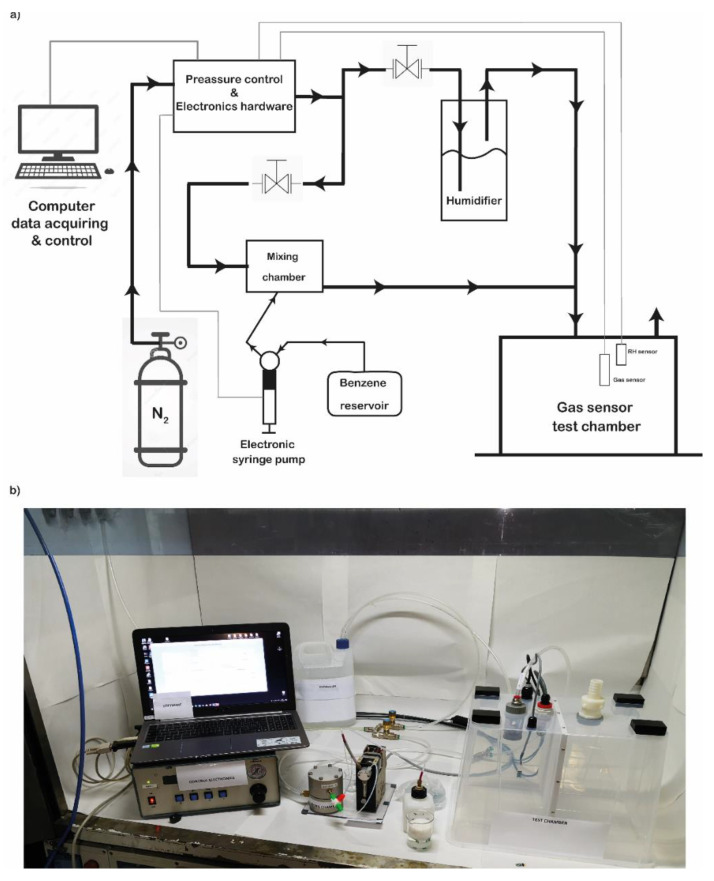
Gas test chamber. Schematic representation of the test chamber (**a**); real picture of the realized and used test chamber (**b**).

We also evaluated the binding of the benzene gas (in gas measurements) to the pOBP functionalized chip by using the gas chamber described in Figure 4.

In order to flux a volume of 1 m^3^ of air, the chips were left under a flow (50 L/min) of nitrogen/benzene mixture for 20 min before the measure was recorded. To obtain the final concentrations of benzene ranging from 64 pM to 1.2 µM, the benzene solution was diluted to the concentrations 0.12 M and 128 µM; the concentrations of benzene at 64 pM, 640 pM, and 1.2 nM were obtained by dispensing benzene at the rates of 25, 250, and 500 µL/min from a 128 µM benzene solution, while for the 1.2 µM benzene concentration value, the 0.12 M benzene solution was used, fluxing at the speed of 500 µL/min. The humidity during the measurements was around 43% and the temperature was 24 °C.

**Figure 5:**
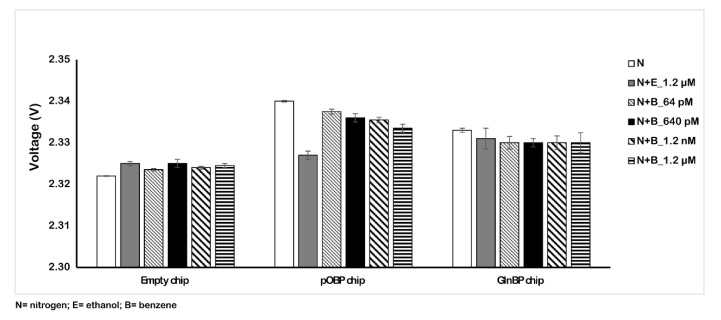
Variation of the voltage value in the absence (nitrogen and ethanol) and in the presence of different concentrations of benzene (from 64 pM to 1.2 µM). The measurements were acquired in gas at room temperature by using the 200 µm sensor chip.

Figure 5 shows the variation of the impedance signal in the absence and in the presence of different benzene concentration values dissolved in the nitrogen flow. The obtained results show the different behavior of the signal as a consequence of the benzene presence in the gas. In particular, in these conditions, we registered a reduction (6 mV) in the voltage value instead of an increase. This effect is due to the dielectric characteristics of the water that influence the signal. The recorded signal reduction was low and similar with respect to the experiments in water and with the blank. In addition, in order to test the specificity of the gas sensing, we tested a solution of 1.2 µM ethanol in water and a nitrogen/ethanol gas mixture. The voltage variation (Table 2) due to the interference is low, confirming the specificity of the pOBP gas sensor.

### 2.2. Benzene Measurement (in Gas) on Interdigitated 75 µm Thin

To increase the performance of the sensor in gas measurements, we designed a new, denser interdigitated gold chip.

In particular, the distance between the interdigitated electrodes was reduced from 200 µm to 75 µm (Figure 1b), allowing us to increase the chip sensitivity in response to the presence of the benzene during the in-gas measurements.

In addition, we optimized the electronics module of the chip to amplify the signal and, consequentially, enhance the sensor sensitivity.

**Figure 6:**
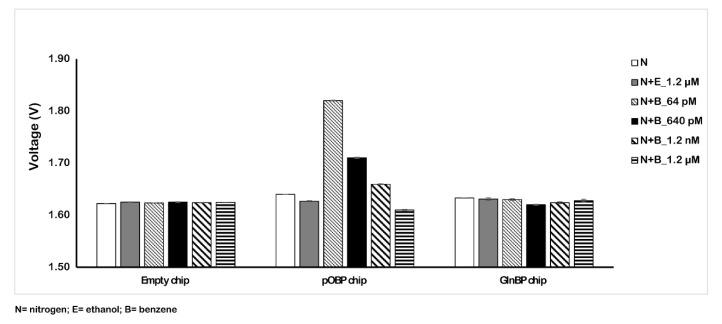
Variation of the voltage in the absence (nitrogen and ethanol) and in the presence of different concentrations of benzene (from 64 pM to 1.2 µM). The measurements were acquired in gas at room temperature by using the 75 µm sensor chip configuration.

Figure 6 reports the obtained results with the amplified 75 µm chip sensor. In particular, testing different concentrations of benzene in the air (from 64 pM to 1.2 µM) on the functionalized pOBP chip, we registered a variation response of the voltage comparable to the response in the absence of benzene (nitrogen and ethanol) and to that with respect to the 200 µm chip.

Table 3 reports the variation values in the impedance signal of the three different samples (nitrogen, ethanol, and benzene gas mixture) on the empty, pOBP, and GlnBP functionalized chip. For the pOBP functionalized chip, the presence of the minimum benzene concentration (64 pM) allowed us to obtain a decrease in the impedance signal that was about 210 mV more marked than the previous chip configuration (6 mV for the 200 µm chip). No significative impedance variation was registered in the presence of ethanol, confirming the specificity for the binding to benzene. Finally, the absence of an impedance signal, in the presence of different benzene concentrations, on the GlnBP chip (negative control) demonstrated the specificity of the functionalized chip with pOBP for benzene gas.

Anyway, due to the number of potential contaminants in real-world applications, it is not possible to exclude the possibility of interferences.

### 2.3. Real Matrix

**Figure 7:**
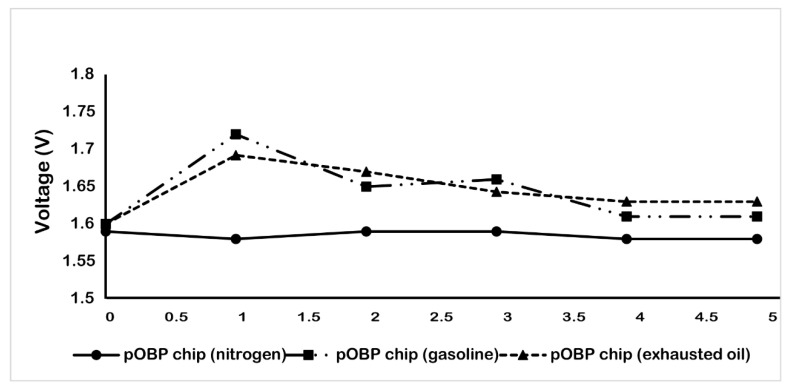
Variation of the voltage, during the time (5 h) in the absence (nitrogen) and in the presence of gasoline and exhausted oil vapor. The measurements were acquired in gas, at room temperature by using the 75 µm sensor chip configuration.

Finally, to evaluate the performance of the developed gas sensor, different impedance measurements were carried out on real matrices (gasoline and exhausted engine oil that contains benzene) that are commonly present in a real scenario (such as in a garage, on a highway, in a refinery, at a petrol station, etc.). The measurements were performed as described in the Materials and Methods section and the obtained data are reported in Figure 7. In particular, the data show that the gas biosensor is able to record the presence of benzene in each of the sample tested. A decrease in the voltage value was observed up to the saturation of the gas sensor, which is highlighted by the presence of a plateau of the curve.

## 3. Materials and Methods

### 3.1. Materials

N-hydroxysuccinimide (NHS), N-(3-dimethylaminopropyl)-N’-ethylcarbodiimide hydrochloride (EDC), butyric acid, ethylenediamine, and α-lipoic acid were purchased from Sigma-Aldrich (Sigma-Aldrich S.r.l., Milan, Italy). All other chemicals were commercial samples of the purest quality.

### 3.2. Expression and Purification of the pOBP

The pOBP was prepared according to Capo A. et al. [5]. To purify the protein, the filtered cellular lysate has been loaded on a GST-trap column installed on an AKTA PURE FPLC system (GE Healthcare), washed and incubated overnight with thrombin enzyme. The eluted protein fractions have been collected, concentrated (five times) with a centrifugal filter device (3000 Da Vivaspin 500 Turbo, Sartorius), and loaded on a gel filtration column (G75 Sephadex). The purity of the protein sample has been verified by SDS-PAGE and the protein concentration has been determined by measuring the absorbance of the sample at 280 nm, considering a molecular weight of 18 kDa and a molar extinction coefficient of 13,000 M^−1^ cm^−1^.

### 3.3. Electrode Preparation and Impedance Measurements

Electroanalytical techniques offer unique access to information on chemical, biochemical, and physical systems. Miniaturized gold-plated electrodes are used and a frequency-dependent vector can describe changes:

Z(jω) = |Z(ω)| exp (jΦz(ω)) ω = 2πf(1)

Impedance measurements are made by applying an alternating sinusoidal voltage to the electrodes and measuring the amplitude and phase shift of the concomitant electrical current that develops across it.

Following this purpose, PCBs equipped with interdigitated electrodes have been developed. In particular, miniaturized gold-plated PCB were produced and tested that are based on standard photolithography technologies. The PCB surfaces have been equipped with interdigitated electrodes with different finger spacings in the range of 75 µm to 200 µm. Even if impedance measurements are often made by applying an alternating voltage in a wide range of frequencies and amplitudes, for analytical applications, it is also possible to limit the measurements to a few or even just one selected frequency. In the experiments, the chosen frequency range was 5 to 15 kHz, as reported in a similar experiment using OBPs performed by Capone et al. [15]. In a second step, to reduce the hardware complexity and consumption, a binary excitation has been preferred. The practical approach to the problem is similar to square-wave voltammetry (SWV—one of the four major voltammetry techniques). The interdigitated electrodes provide a suitable tool that is especially useful for impedance, capacitance, and conductivity measurements. The electronics through different development phases are now running in a very low-energy consumption configuration.

In ordinary conditions, the sensor will have a relatively long life, as complete saturation of the pOBP molecules may derive only from a massive and rare event. The sensing unit has the specifications reported in Table 4.

### 3.4. Gas Chamber Design and Setup

The electric impedance (EI) measuring system has been tested in a chamber connected to a gas-mixing bench in order to evaluate the sensitivity to the target gas. The characterization of the OBP binding of benzene has been performed in controlled conditions (fixed gas flow and regulated temperature and humidity).

The gas sensor chamber was developed with the aim to expose the chip to controlled atmospheres of standard gas mixtures of the selected pollutants and to verify the capability to bind the selected molecules in the gas phase. The concentrations of the target gas are obtained with a suitable dilution in a reference air or nitrogen flow. Stock solution of 0.18% C_6_H_6_ were used in a PBS buffer at pH 8.3 in a PC-controlled syringe pump. The concentration ranges from 0.1 up to 10 mg/m^3^. The detection limit was fixed to 0.5 mg/m^3^. The gas sensor has been exposed to different concentrations of benzene, gasoline, and oil, and the variation in voltage signals was monitored in real time.

### 3.5. Surface Derivatization and Functionalization

The gold interdigitated surface before the derivatization and functionalization were washed three times with MilliQ water and dried under nitrogen flow. The gold IDEs was functionalized according to the derivatization protocol by Cennamo et al. [14]. In brief, the cleaned chips were first incubated overnight at 25 °C in 40 mM α-lipoic acid dissolved in 10% ethanol to allow the formation of a thiol self-assembled monolayer (SAM) on the gold surface, and then the carboxylic moiety of α-lipoic acid was activated with a mixture of EDC 200 mM and NHS 50 mM in 20 mM sodium phosphate buffer, pH 7.0, for 15 min. In the final step, 15 µL of a 2 mg/mL pOBP sample (or glutamine-binding protein, used as a negative control) were deposited on the gold surfaces and incubated for 2 h at room temperature. To improve protein immobilization performance, the second steps of the above protocol have been repeated so as to improve the active MREs density on the surface of the IDE. A similar procedure was performed in blank chip preparation, and for this purpose, we selected the glutamine-binding protein (GlnBP) isolated from *E. coli* for the immobilization.

### 3.6. Benzene Measurements

The benzene detection measurements were carried out in both (i) liquid and (ii) gas configurations.

(i) In liquid measurements, the functionalized surface was incubated in the absence and presence of an increased concentration of benzene diluted in the PBS buffer. The measurements were performed at room temperature, and each sample was incubated for 10 min before acquiring the voltage value.

(ii) Under the same experimental condition, benzene measurements were performed in the gas sample. The testing system allows different concentrations of gas and/or volatiles to pass through using a suitable dilution of the related analytes in a reference nitrogen flow. The real (ReZ) and imaginary (ImZ) parts of Z describe the resistance (R) and reactance (X), which can be represented by an appropriate electrical circuit in series. In this case, three different steps were performed:

1. Drying the air inside the gas chamber;
2. Exposition of each sample in the gas chamber under a constant flow of humid air in the absence and in the presence of an increasing concentration of benzene;
3. Impedance data were collected stepwise in discrete intervals following the evolution of the system up to a steady state.

### 3.7. Real Matrix Measurements

Measurements on real matrix samples were performed as follows: 50 mL of gasoline and/or exhausted engine oil (heated up to 50 °C) were placed in the gas sensor test chamber for 1 h, until the chamber were completely saturated with the vapour. The gas sensor was placed in the test chamber and the measurements were carried out for 5 h, registering the voltage value each hour.

### 3.8. Firmware and Software Configuration

Firmware and software were developed to control the gas sampler, the humidity, the reference gas analyzer, benzene supplier, and to acquire the output voltage from the developed sensor.

The software was developed using the C# programming language in Visual Studio 2017 (Microsoft, Redmond, WA, USA), which supervises the airflow and the benzene release inside the gas chamber while continuously monitoring the environmental conditions, i.e., temperature and RH. A proportional controller (with Kp = 0.1) was employed for airflow regulation inside the chamber, allowing us to manually adjust the flow rate set point in the interval spanning from 10 to 60 L/min. Regarding the benzene release, the software controls a syringe pump (CAVRO XP3000, Tecan, Männedorf, Switzerland), allowing for (1) the manual control of benzene flow during the priming operation of the measurement chamber; (2) the activation of an automatic benzene dispersion, by setting the desired flow rate in the range of 25–500 µL/min. The data acquisition was performed through a National Instruments (National Instruments, Austin, TX, USA) USB 6003, and it is then possible to select the sampling frequency (100–1000 Hz). Data are sampled and filtered (20 points moving average filter) and shown in a dedicated plot. Additionally, continuous measurements of the temperature are provided in real time. The used sensors for temperature and RH and airflow (benzene and nitrogen) were, respectively, HTM 2500 LF (Measurement Specialties, Toulouse, France) and AWM 5104 VN (Honeywell).

### 3.9. Statistical Analysis

Each measurement was performed as triplicate. From the value of the triplicates, the mean and standard deviation were calculated. The graphs reported the mean values gleaned from the blank values, and the values of the error bar represented the calculated standard deviation. The graphs were realized in Excel 2016 by Microsoft^®^ and/or in Origin Pro 8.0 software.

## 4. Conclusions

In conclusion, in this work, we have showed the possibility of using the pOBP as a probe for the low-energy impedenziometric detection of the presence of benzene for outdoor and real-time analysis in air. The developed assay, used as a first-line detection, is robust and, in addition, it is able to detect the presence of benzene at a concentration of 64 pM (5 µg/m^3^), which represents the limit value set by the European Directive 2008/50/EC on human health protection. We plan to optimize the chip setup to perform quantitative measurements.

## Figures and Tables

**Table 1:** Variation of the voltage of 200 µm interdigitated sensor chip in three different conditions.

	200 µm Sensor Chip						
	A	W	W+E_1.2 µM	W+B_64 pM	W+B_640 pM	W+B_1.2 nM	W+B_1.2 µM
**Empty chip**	2.159 ± (0.010)	2.322 ± (0.013)	2.280 ± (0.010)	2.295 ± (0.002)	2.286 ± (0.001)	2.304 ± (0.003)	2.282 ± (0.009)
**pOBP chip**	2.159 ± (0.010)	2.408 ± (0.011)	2.320 ± (0.012)	2.390 ± (0.001)	2.430 ± (0.001)	2.512 ± (0.002)	2.546 ± (0.010)
**GlnBP chip**	2.159 ± (0.010)	2.401 ± (0.016)	2.300 ± (0.013)	2.287 ± (0.001)	2.290 ± (0.002)	2.292 ± (0.010)	2.301 ± (0.008)
**pOBP—GlnBP**	0.000 ± (0.020)	0.007 ± (0.027)	0.020 ± (0.025)	0.103 ± (0.002)	0.140 ± (0.003)	0.220 ± (0.012)	0.245 ± (0.018)

A = air; W = water; E = ethanol; B = benzene.

**Table 2:** Variation of the 200 µm sensor chip voltage in three different conditions.

	200 µm Sensor Chip					
	N	N+E_1.2 µM	N+B_64 pM	N+B_640 pM	N+B_1.2 nM	N+B_1.2 µM
**Empty chip**	2.322 ± (0.001)	2.325 ± (0.002)	2.324 ± (0.000)	2.325 ± (0.003)	2.324 ± (0.001)	2.325 ± (0.001)
**pOBP chip**	2.340 ± (0.002)	2.327 ± (0.003)	2.338 ± (0.001)	2.336 ± (0.004)	2.336 ± (0.001)	2.334 ± (0.003)
**GlnBP chip**	2.333 ± (0.001)	2.331 ± (0.009)	2.330 ± (0.006)	2.330 ± (0.003)	2.330 ± (0.006)	2.330 ± (0.009)

N = nitrogen; E = ethanol; B = benzene.

**Table 3:** Variation of the 75 µm sensor chip voltage in three different conditions.

	75 µm Sensor Chip					
	N	N+E_1.2 µM	N+B_64 pM	N+B_640 pM	N+B_1.2 nM	N+B_1.2 µM
**Empty chip**	1.622 ± (0.000)	1.625 ± (0.001)	1.624 ± (0.001)	1.625 ± (0.001)	1.624 ± (0.001)	1.625 ± (0.001)
**pOBP chip**	1.640 ± (0.000)	1.627 ± (0.001)	1.820 ± (0.001)	1.710 ± (0.001)	1.660 ± (0.001)	1.610 ± (0.001)
**GlnBP chip**	1.633 ± (0.001)	1.631 ± (0.003)	1.630 ± (0.002)	1.620 ± (0.001)	1.624 ± (0.002)	1.628 ± (0.002)
**pOBP—GlnBP**	0.007 ± (0.001)	-0.004 ± (0.004)	0.190 ± (0.003)	0.090 ± (0.002)	0.036 ± (0.003)	−0.018 ± (0.003)

N = nitrogen; E = ethanol; B = benzene.

**Table 4:** Sensing unit electronic specifications.

Interdigitated Electrodes	Power Consumption	Analog Output
200 and 75 μm	I < 1.2 mA	Ov: 0.5–2.5 Volt
Gold-plated PCB	NsV: 3.3 Volt	LPF: 3 Hz
Waterproof	OsV: 3~6 Volt	RefV: 1.80 Volt
Dust proof		VCO: 11 kHz (5 to 50 kHz)
Particle proof		OpT: 0~30 °C

I = Current; NsV = nominal supply voltage; OsV = operation supply voltage; Ov = output voltage range; LPF = low-pass filter; RefV = reference voltage; VCO = oscillator (VCO); OpT: operation temperature range.

## Data Availability

Not applicable.
